# Combined Role of Fe Nanoparticles (Fe NPs) and *Staphylococcus aureus* L. in the Alleviation of Chromium Stress in Rice Plants

**DOI:** 10.3390/life12030338

**Published:** 2022-02-24

**Authors:** Hesham F. Alharby, Shafaqat Ali

**Affiliations:** 1 Department of Biological Sciences, Faculty of Science, King Abdulaziz University, Jeddah 21589, Saudi Arabia; halharby@kau.edu.sa; 2 Department of Environmental Science and Engineering, Government College University, Faisalabad 38000, Pakistan; 3 Department of Biological Sciences and Technology, China Medical University, Taichung 40402, Taiwan

**Keywords:** chromium, physiological parameters, oxidants, antioxidants, rice, heavy metals

## Abstract

Chromium (Cr) is a toxic heavy metal whose high concentration in soil badly affects plant growth, photosynthesis, and overall yield. Metal-derived nano-particles and metal-resistant bacteria can strengthen the plant defense system against different abiotic stresses; however, little is known about the use of nanoparticles in conjunction with bacteria. This study examined the combined effect of Fe nanoparticles (Fe NPs) and a chromium-resistant bacterium *Staphylococcus aureus,* on rice plants grown on chromium saturated medium. Chromium stress reduced rice growth, biomass, and chlorophyll contents by causing oxidative damage leading to overproduction of electrolyte leakage, hydrogen peroxide, and malondialdehyde. Fe NPs significantly improved plant growth, biomass, yield, and photosynthetic activity by enhancing the chlorophyll contents and alleviating oxidative damage. Application of Fe NPs also reduced the uptake and accumulation of Cr in the plants by increasing the bioavailability of micronutrients to the plant. The Fe NPs decreased oxidative damage and enhanced the enzymatic and non-enzymatic activity in the plant to withstand Cr stress compared to the plants without Fe NPs treatments. The inoculation of rice plants with the chromium-resistant bacteria *S. aureus* further enhanced the positive impact of Fe NPs by transforming the toxic form of chromium (Cr^6+^) into a less toxic form of chromium (Cr^3+^). The bacterial inoculation reduced Cr uptake by plants through adsorption of Cr ions, resulting in decreased chromium ion bioavailability. At chromium level 100 mg/kg, the foliar application of Fe NPs from 0 to 20 mg/L increased the total chlorophyll contents from 2.8 to 3.9. The application of *S. aureus* further enhanced the chlorophyll contents from 4.4 to 5.4, respectively. The current study suggested that combining Fe NPs and *S. aureus* could be a viable strategy for reducing Cr toxicity and accumulation in rice plants and most likely other plants.

## 1. Introduction

Soil pollution, caused by the discharge of toxic metals, has become a major environmental issue worldwide. Chromium (Cr) is a toxic metal element whose chemical compounds are widely distributed in the atmosphere due to industrial waste, primarily tannery effluents, metallurgy, and rapid urbanization [1,2]. When aggravated, Cr compounds reach our food chains; their high persistence and soil availability can be absorbed or stored in plants (directly or indirectly), thus causing harmful effects on human health when the latter are consumed [3]. Phytotoxic effects are known to be extreme in plant species grown in Cr-contaminated areas. The toxic effect and cancerous properties of chromium (VI) have been renowned for a long time. Water-insoluble chromium (III) compounds and chromium metal, on the other hand, are not considered harmful [2]. Chromium can easily travel to various plant parts and can be accumulated there before being consumed by animals and humans [4]. Chromium harms plants by reducing plant height and root growth, interrupting germination, unbalancing nutrient levels, and causing photosynthesis damage [5]. Further chromium exposure slows soil microbial activity, inhibits enzyme activity, and triggers the formation of reactive oxygen species (ROS), leading to plant oxidative stress [6]. Chromium can lead to many problems in human biological systems, including death in those exposed to it.

Rice (*Oryza sativa* L.) is the most commonly consumed cereal grain, with over half of the world population eating it as a staple meal. After sugarcane and maize, it is the agricultural commodity with the third-highest global output (741.5 million metric tons). It is grown in more than 100 countries worldwide, accounting for 21% of the world’s total caloric intake and up to 76% of the population of Southeast Asia [7]. Rice is high in carbohydrates, which serve as fuel for the body. It also contains fiber, which aids digestion and lowers cholesterol and saturated fats, making it a heart-healthy meal [8]. The pollution of paddy rice by carcinogenic and harmful contaminants such as heavy metals has risen due to increased industrial growth, with the potential to imperil the entire food chain and, as a result, human health [9,10]. Rice is grown in flood-prone areas, which encourages the plant’s mobilization and eventual accumulation of heavy metals [11,12]. The indiscriminate use of chemical products and waste originating from industry and agriculture are all main contributors to the rise and mobilization of toxic metals in rice plant tissues [12]. The four main processes for heavy metal accumulation in rice are root absorption, root-to-shoot translocation, redirection at nodes, and remobilization from leaves [13]. Cadmium (Cd), chromium (Cr), lead (Pb), mercury (Hg), and arsenic (As) are among the heavy metals that are toxic to both plants and humans [14]. Hexavalent Cr (Cr^6+^) is more toxic than trivalent chromium (Cr^3+^) due to an elevated oxidation state. Toxicity of Cr^6+^ interferes with various physiological functions in plants, including rice, resulting in a reduction in seed germination, development, and photosynthesis and increased changes in leaf protein profiles and root microRNA expression [15,16]. Previous studies found that induction of oxidative stress was the key biological mechanism underlying Cr toxicity in plants, based on the effect of Cr exposure on the roots of growing rice seedlings [17]. The activation of CDPK (calcium-dependent protein kinase) and NADPH (nicotinamide adenine dinucleotide phosphate) oxidase in response to Cr (VI) inhibits rice root growth by accumulating calcium and producing reactive oxygen species, respectively [18].

Nanotechnology is one of the promising scientific fields that have the potential to revolutionize agricultural production [19,20]. Biofortification of micronutrients is the process of enriching the nutrient content of crops to provide a long-term solution to nutrient deficiencies in agricultural products, mainly Fe and Zn deficiency [21]. The size, shape, reactivity, chemical compositions, and surface functionalization of various engineered NPs decide their performance [19,22]. The properties of these transition metal NPs and their growing applications in agriculture, biology, and biomedicine have prompted increased interest in their synthesis [23,24]. Micronutrients, especially Fe, play an essential role in various physiological, metabolic, and cellular processes in plants due to their ability to promote oxidation and reduction reactions [25]. Nanoparticles have been shown to affect microscopic soil properties such as humic acid content, bacterial communities, and plant growth and development [26]. Depending upon the properties of nanoparticles, the plants and NPs interaction may positively and negatively impact the plant’s physiological, morphological, and molecular features [27]. The nanoscale metals and metal oxide may damage or improve the germination, plant growth, development, photosynthesis, and yield parameters [26]. Many studies reported that the application of iron oxide nanoparticles increased the germination, improved photosynthesis, reduced drought and metal stress and increased nutrients absorption, phytohormones production, and crops yield in maize, cotton, wheat, rice, tomato, and cereal crops [27,28,29].

Microorganisms play a crucial role in mineralizing pollutants, including heavy metals and the biogeochemical transformation of nutrients in the soil [30]. Microbes can reduce metal accumulation in plants by altering the redox state of metal species in the soil, resulting in increased plant growth and biomass production. Chromium-reducing bacteria can reduce Cr^6+^ to Cr^3+^ in the rhizosphere through bioaccumulation and biosorption mechanisms, reducing Cr toxicity [31]. *Staphylococcus aureus* is a gram-positive, round-shaped, and chromium-reducing bacterium first-time isolated from tannery effluent [32].

The metals’ nanoparticles and bacteria can promote plant growth and reduce the stress of metals’ toxicity in plants. The combined role of nanoparticles and bacteria in alleviating metal-induced toxicity in plants has not been studied thoroughly. This study aimed to analyze the combined and individual roles of the chromium-reducing bacterium Staphylococcus aureus and Fe NPs to alleviate the chromium-induced (Cr^6+^) toxicity in rice plants.

## 2. Materials and Methods

### 2.1. Soil Sampling and Analysis

The soil for this experiment was obtained from the field of the University of Agriculture, Faisalabad-Pakistan. The soil was sieved through a 2 mm sieve, and large debris was removed thoroughly. Initial soil characterization was accomplished by a standard procedure such as particle size by hydrometer [33]. The electrical conductivity (EC) of saturated soil paste was measured by a calibrated EC meter. Similarly, the pH of the soil paste with a 1:25 soil to water ratio was noted by calibrated pH meter. The soil was treated with ammonium bicarbonate diethylene triamine penta acetic acid (AB-DTPA) at pH 6.7 to analyze the available trace elements in the selected soil [34]. The standard procedure was used to determine pseudo total metals in the soil [35], which is described in Table 1.

### 2.2. Seed Inoculation

Seeds of the rice were inoculated with chromium-resistant bacteria. For this purpose, peat moss as a source of bacterial inoculation was obtained from the Ayub Agriculture Research Institute, Faisalabad, Punjab, Pakistan. The soil was dried adequately at 70 °C for 72 h, then grounded and sieved through a 2 mm sieve and autoclaved at 121 °C for 20 min to eliminate bacterial contamination. The bacterial inoculum was synthesized by nutrient broth and Cr-resistant bacteria (*Staphylococcus aureus*), added to the inoculum, and shaken at 2000 rpm for 48 h at 30 °C for 48 h. The density of bacterial isolates was measured by hemocytometer. After 48 h of shaking, the inoculum was added into the conical tubes from the flasks and centrifuged at 6000 rpm for 10 min. The supernatant was removed and diluted with distilled water to re-suspend the pellets [36]. The population size of centrifuged bacterial cells was adjusted at 2.8 × 10^8^ wet weight. The seeds of rice were disinfected by inoculum with a 10% sugar solution. Then, the seeds were adequately coated with clay, and peat moss took in equal amounts (1:1) and was placed overnight.

### 2.3. Pot Experiment

A pot experiment was set up at the botanic yard of Government College University, Faisalabad-Pakistan, at a temperature of 18–25 °C with a humidity of 70%. Sieved soil was mixed with various chromium concentrations (0, 50, and 100 mg/kg) using K_2_Cr_3_O_7_ as Cr^6+^ source. Each plastic container was filled with 5 kg of soil, and five rice seeds were sown in each pot. Thinning was performed after germination, and only two healthy seedlings were preserved. For healthy growth, nitrogen, phosphorus, and potassium were applied at 120:50:2 Kg/ha. Plants were sprayed with varying concentrations of Fe nanoparticles (0, 10, and 20 mg/L) after two weeks of germination, while controls were treated with filtered water. Fe NPs was purchased from Alfa Aesar. The characterization revealed the purity of Fe NPs (Fe_3_O_4_. Iron (II, III) oxide) was 97%, size 50–100 nm and density 5.2 and also used in our previous study [37].

### 2.4. Growth and Physiological Parameters

After four months of treatment, plants were harvested by chopping the shoots around 1 cm high from the soil. The harvested plants were cut into various sections, washed, and dried in the oven for 72 h at 70 °C, and their dry weight was measured. Roots were washed with 1% HCl and rinsed properly with filtered water to ensure the complete removal of acid, and dry weight of roots were noted.

Growth parameters such as plant height (cm), root and shoot length (cm), and spike length (cm) were measured by meter scale. The number of leaves per plant was counted, and the fresh and dry biomass of root and shoot was measured by weighing balance. Chlorophyll a, b, total chlorophyll, and carotenoids contents were determined by a method followed by Lichtenthaler [38]. To quantify the chlorophyll contents, the samples were extracted in 85% acetone and 15% distilled H_2_O_2_ (*v*/*v* ratio) and were centrifuged, and readings were taken using a spectrophotometer at specified (645, 480, and 663 nm) wavelengths.

### 2.5. MDA, EL, H_2_O_2_, and Antioxidants Enzymes

Malondialdehyde (MDA) content was determined by the procedure described by Zhang and Kirham [39]. Electrolyte leakage (EL) was measured by the Dionisio-Sese and Tobita [40] method. First, samples were extracted in trichloroacetic acid (TCA) solution at 32 °C for 2 h, after which the first EC of the solution was recorded. Then, the procedure was repeated at 121 °C for 20 min, after which the final EC of the solution was documented. Following Jana and Choudhuri’s [41] procedure, the contents of H_2_O_2_ were estimated. These samples were homogenized before centrifugation by adding 50 Mm phosphate buffer at pH 6.5, then centrifuged for 20 min. After that, H_2_SO_4_ (20% *v*/*v*) was added to the ultra-spin separated mixture and centrifuged for another 15 min. At a wavelength of 410 nm, the intake was detected. For an estimate of POD and SOD contents, specimens were macerated in liquid nitrogen and then systemized in phosphate buffer with 0.5 M at 7.8 pH [42]. APEX activities were assessed using the Nakano and Asada [43] technique, while CAT activity was calculated using the Aebi [44] method.

### 2.6. Metal Contents

In order to determine the amount of iron (Fe) and chromium (Cr) in roots, shoots, and grain samples, 1 g of each sample was processed in a heated plate with a 4:1 ratio of HNO_3_: HClO_4_ (*v*/*v*). The amount of metal was measured in processed samples using an atomic absorption spectrophotometer (AAS) [45].

### 2.7. Statistical Analysis

ANOVA was used to assess statistics using SPSS (Statistics Programming, Version 21) at a probability level of 5 percent. For various method differentiations, Tukey’s HSD post hoc test was performed.

## 3. Results

### 3.1. Growth Parameters

**Figure 1:**
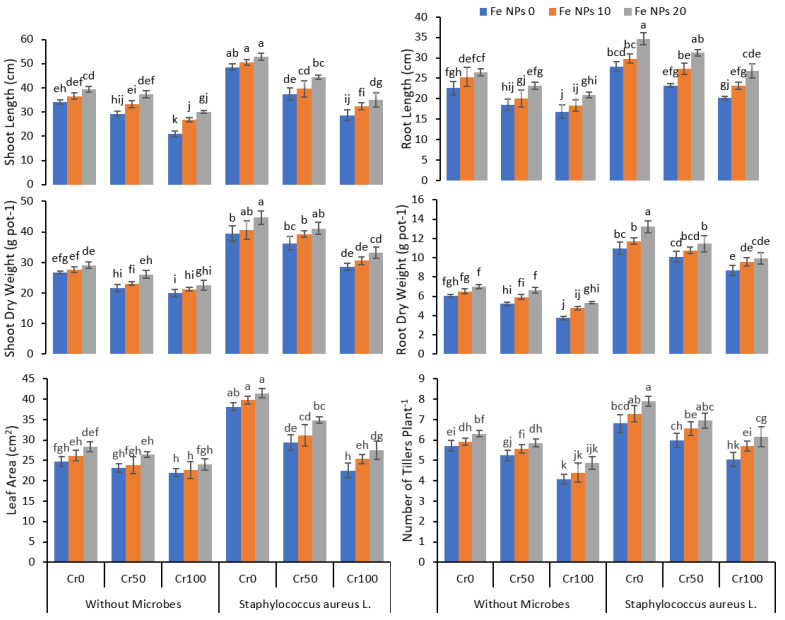
Combined effects of Fe nanoparticles (NPs) (0, 10, and 20 mg L^−1^) and *S. aureus* L. on growth parameters of rice grown under Cr stress (0, 50, and 100 mg kg^−1^). The mean values of three replicates together with their standard deviation are shown in the plots. Various small letters denote significant differences between different treatments at *p* ≤ 0.05.

The application of Cr drastically affected the growth of the rice plants (Figure 1). The growth was gradually decreased when the Cr concentration in the growth medium increased from 25 to 100 mg kg^−1^. The high concentration of Cr 100 mg kg^−1^ caused a significant reduction in the length of the shoot, roots, shoot and root dry weight, number of tillers, and leaf area. Fe NPs had a significant impact on plant growth and improved plant growth even at a high chromium concentration compared to plants without Fe NPs. The increasing concentration of Fe NPs from 0 to 20 mg L^−1^ significantly improved plant growth. The inoculation of *S. aureus* L. in combination with Fe NPs further enhanced the plant growth and reduced the effect of chromium toxicity on plants compared to plant without Fe NPs and bacterial inoculation. The data regarding plant growth attributes indicated that the combined application of *S. aures* and Fe NPs significantly improved the rice growth and dry biomass under Cr stress conditions.

### 3.2. Chlorophyll Contents

**Figure 2:**
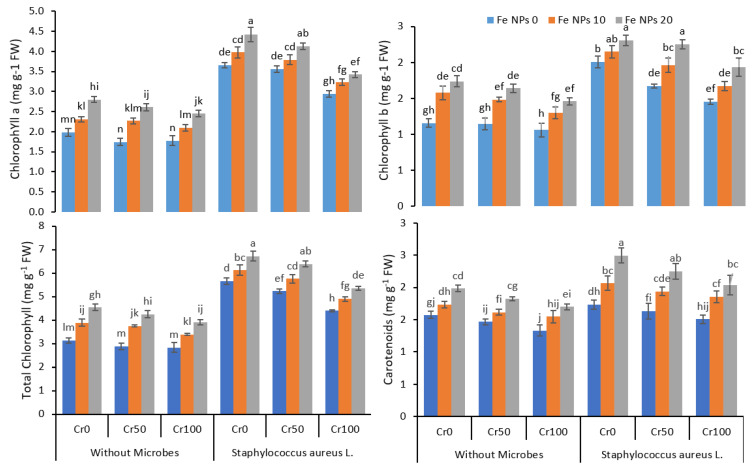
Combined effects of Fe nanoparticles (NPs) (0, 10, and 20 mg L^−1^) and *S. aureus* L. on chlorophyll and carotenoids of rice grown under Cr stress (0, 50, and 100 mg kg^−1^). The mean values of three replicates together with their standard deviation are shown in the figure. Various small letters denote significant differences between different treatments at *p* ≤ 0.05.

The increasing concentration of Cr gradually decreased chlorophyll a, chlorophyll b, total chlorophyll, and carotenoids concentration (Figure 2). The increasing concentration of Cr from 0 to 100 mg kg^−1^ reduced the chlorophyll contents from 2 mg g^−1^ FW to 1.8 mg g^−1^ FW in treatments without microbes and Fe NPs application. The application of Fe NPs (0 to 20 mg kg^−1^) improved the chlorophyll contents from 1.8 to 2.4 mg g^−1^ FW despite increasing Cr from 0 to 100 mg kg^−1^. The inoculation coupled with Fe NPs further enhanced the chlorophyll a, chlorophyll b, and total chlorophyll and carotenoids contents in the rice plants under chromium stress.

### 3.3. IRGA Parameters

**Figure 3:**
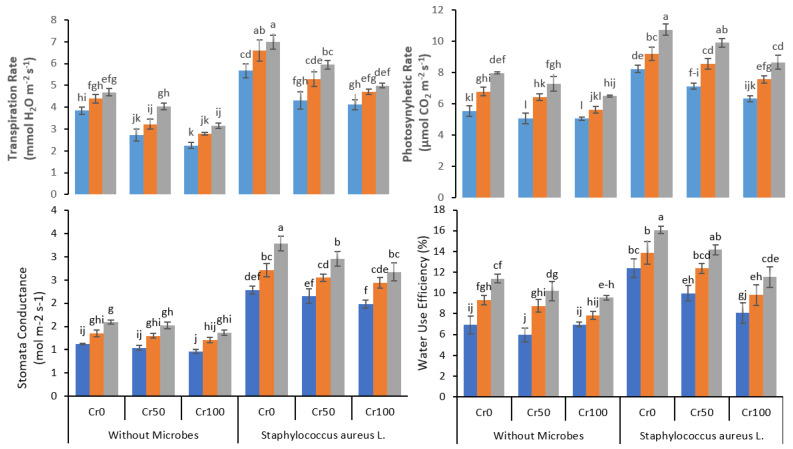
Combined effects of Fe NPs (0, 10, and 20 mg L^−1^) and *S. aureus* on gas exchange attributes of rice grown under Cr stress (0, 50, and 100 mg kg^−1^). The mean values of three replicates together with their standard deviation are shown in figure. Various small letters denote significant differences between different treatments at *p* ≤ 0.05.

The increased Cr concentration steadily decreased the transpiration rate, photosynthetic rate, stomatal conductance, and water use efficiency (Figure 3). The application of Fe NPs significantly improved the transpiration rate, photosynthetic rate, stomatal conductance, and water use efficiency, further boosted by bacterial inoculation in Cr stressed plants. In plants under 100 mg kg^−1^ Cr stress, treated with 20 mg kg^−1^ Fe NPs with no bacterial inoculation, the transpiration rate increased from 3.2 to 5 after bacterial inoculation. At Cr 100mg kg^−1^ and Fe NP 20mg kg^−1^, a similar increase was observed in photosynthetic rate from 6.5 to 8.7 (μmol H_2_O m^−2^ s^−1^), stomatal conductance from 1.4 to 2.7 (mol m^−2^ s^−1^), and water use efficiency from 9.5% to 11.5% after bacterial inoculation.

### 3.4. Estimation of EL, MDA, and H_2_O_2_

**Figure 4:**
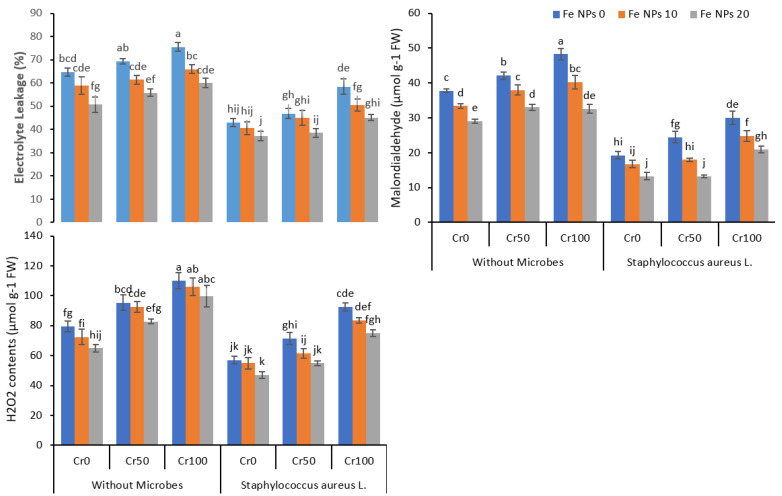
Combined effects of Fe NPs (0, 10, and 20 mg L^−1^) and *S. aureus* on oxidative stress indicating parameters of rice grown under Cr stress (0, 50, and 100 mg kg^−1^). Mean values of three replicates together with its standard deviation are shown in the figure. Various small letters denote significant differences between different treatments at *p* ≤ 0.05.

**Figure 5:**
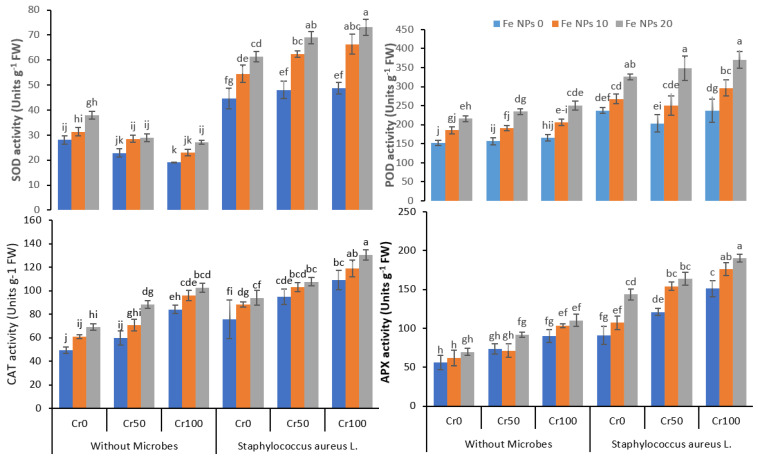
Combined effects of Fe NPs (0, 10, and 20 mg L^−1^) and *S. aureus* L. on antioxidants enzymatic activities of rice grown under Cr stress (0, 50, and 100 mg kg^−1^). The mean values of three replicates together with their standard deviation are shown in the figure. Various small letters denote significant differences between different treatments at *p* ≤ 0.05.

Chromium stress resulted in a substantial increase in electrolyte leakage in rice plants (Figure 4). Under all chromium levels (0, 25, 50, and 100 mg kg^−1^), plants without Fe NPs treatment showed more EL than plants provided with Fe NPs. Similarly, non-inoculated plants showed more EL as compared to inoculated plants. The combined application of Fe NPs and *S. aureus* L. significantly reduced the EL in rice plants at all chromium levels. As evident in Figure 5, Fe NPs (0 to 20 mg kg^−1^) and *S. aureus* L. reduced the EL from 58.4% to 45.1% at chromium level at 100 mg kg^−1^.

Similarly, Cr toxicity also increased the lipid peroxidation, and a noticeable increase was observed in MDA contents in leaves of rice plants. Maximum MDA contents were observed in plants without Fe NPs and bacterial inoculation under chromium stress at 50 and 100 mg kg^−1^. Fe NPs reduced the MDA contents in plants under different levels of chromium stress, further decreasing by combining Fe NPs and S. aureus. Likewise, a gradual rise in H_2_O_2_ was observed in plants under different concentrations of chromium. After the Fe NPs and *S. aureus* L. application individually and combined, a noteworthy decrease was observed in H_2_O_2_ content in plants under Cr stress.

### 3.5. Anti-Oxidant Enzymes Activities

The findings revealed that SOD activity was significantly increased with the increasing concentration of chromium. The application of Fe NPs enhanced the increase in SOD activity, further boosted by the combined application of Fe NPs and bacterial inoculation. In the plants under chromium stress (100 mg kg^−1^), the SOD activity increased from 48.7 g^−1^ FW (Fe NPs 0) to 73.1 (Fe NPs 20 mg kg^−1^) coupled with bacterial inoculation. Similarly, POD activity also improved from 236.1 to 370.8 g^−1^ FW by combining Fe NPs and *S. aureus* L. in plants treated with 100 mg kg^−1^ of chromium. Likewise, an increase was observed for CAT and APX activity after combining Fe NPs and *S. aureus* L. The CAT and APX activities increased from 109.1 to 130.5 g^−1^ FW and 151.3 to 190.2 g^−1^ FW, respectively, by increasing Fe NPs from 0 to 20 coupled with bacterial inoculation chromium level 100 mg kg^−1^.

### 3.6. Chromium Uptake by Plants

**Figure 6:**
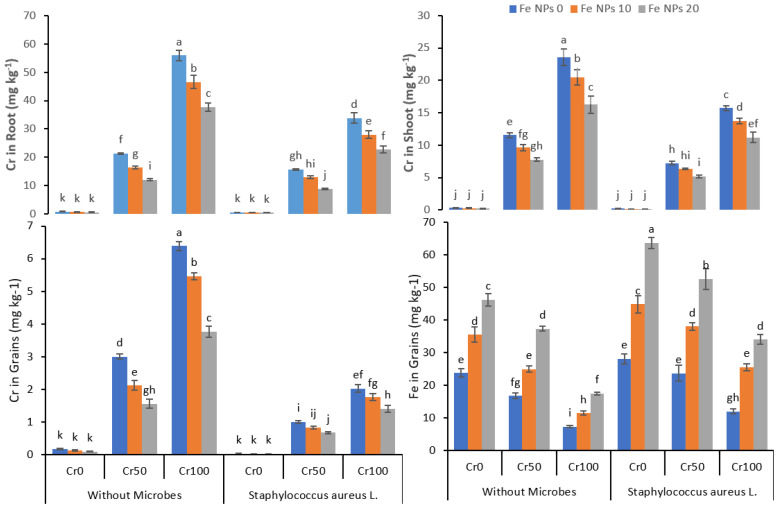
Combined effects of Fe NPs (0, 10, and 20 mg L^−1^) and *S. aureus* L. on Cr concentrations in roots, shoot, grains, and Fe concentration in grains of rice grown under Cr stress (0, 50, and 100 mg kg^−1^). Mean values of three replicate together with its standard deviation are shown in figure. Various small letters denote significant differences between different treatments at *p* ≤ 0.05.

The data regarding chromium accumulation in the root, shoot, and grains and Fe accumulation in grains are shown in Figure 6. The increasing chromium concentration from 0 to 100 mg kg^−1^ also increased the chromium accumulation in the root, shoot, and grains in treatments without Fe NPs and bacterial inoculation. The combined application of Fe NPs and *S. aureus* L. significantly reduced the Cr uptake and accumulation in the plants. On the other hand, the increasing concentration of Cr from 0 to 100 mg kg^−1^ reduced the iron accumulation despite increasing Fe NPs concentration from 0 to 20 mg kg^−1^ both in non-inoculated and inoculated rice plants.

## 4. Discussion

In this study, rice plants were cultivated in Cr-contaminated soil treated with foliar application of Fe NP coupled with microbes to determine the role of Fe NPs and S. *aureus* L. in alleviating the Cr induced toxicity in rice plants. According to the findings, the plant growth was significantly reduced under Cr stress without foliar application of Fe NPs and/or *S. aureus* L. These findings are consistent with previous research that has found Cr to be toxic to various plant species [3,5,46,47]. This disruption in rice growth could be attributed to Cr’s adverse effects on plant morphology, physiology, and mineral accumulation [47]. The hexavalent chromium (Cr^6+^) high concentration in plants may restrict the cell division, thus limiting the plant growth, especially roots. It was evident from the results that foliar application of Fe NPs caused a significant positive impact on plants growth. The Fe NPs increased the root and shoot length, root and shoot dry weight, the number of tillers per plant, and leaf area of the rice plants under Cr stress. It has previously been well-demonstrated that nanoparticles have great potential for supplying nutrients to the plants [48,49].

Furthermore, the nanoparticles can activate the metabolic system of the plants, which is beneficial for plant growth and development [49]. Additionally, the role of Fe NPs on plant growth might be attributed to the ability of Fe to stimulate the biosynthesis of chlorophyll and redox process in the plant, which may boost the plant growth [37]. The inoculation of plants with *S. aureus* L. significantly improved all growth parameters in plants under Cr stress. The chromium-resistant bacteria *S. aureus* L. can reduce the Cr^6+^, a highly toxic form of chromium, to Cr^3+^, a less toxic form for plants. The improvement in the plants’ growth parameters after inoculation may be linked to the ability of bacteria to detoxify the Cr^6+^ by their intracellular, enzymatic, and metabolic process [50]. The production of waste such as H_2_S by bacteria also contributes to detoxifying the Cr hexavalent form of chromium to the trivalent form of chromium [51].

Chlorophyll is a significant component of chloroplasts and is related to plant photosynthetic rate. Any changes in chlorophyll contents can indicate plant health and plant response to environmental stress [4,52]. Different environmental stress can decrease the chlorophyll contents in the leaves of the plants [52]. The Cr-stress drastically reduced the chlorophyll a, chlorophyll b, total chlorophyll, and carotenoids contents in rice plants (Figure 2). Similarly, the only treatment of plants with Cr reduced the plant transpiration rate, photosynthetic rate, stomatal conductance, and water use efficiency (Figure 3). The application of Fe NPs reversed the decrease in the chlorophyll and carotenoids contents induced by Cr toxicity. The Fe NPs have been reported to increase the availability of water and nutrients to the stressed plants, thus improving the plants’ physiological performance. Nanoparticles such as iron oxide nanoparticles have been reported to increase the chlorophyll contents in rice plants under Cd stress [48,53]. *S. aureus* L. further improved the chlorophyll contents in the plants under Cr-stressed by their ability to detoxify the hexavalent chromium into trivalent from their metabolic process. The findings suggest that inoculated bacteria may enhance the plant’s ability to withstand the stress of the metal reported earlier by many researchers [54,55]. Other researchers reported similar findings where inoculation of plants with metal-resistant bacteria improved the leaf chlorophyll contents by limiting the metals’ availability to plants and detoxifying the metals [4].

Fe NPs and bacterial inoculation application also significantly improved the transpiration rate, photosynthetic activity, stomatal conductance, and water use efficiency. The high chlorophyll content from Fe NPs and bacterial inoculation improved the overall photosynthetic process and water use efficiency in rice plants [18].

The exposure of the plants to metal stress may disturb the antioxidant enzymes activities, and it may enhance the oxidative stress in the plants [56]. In this study, the Cr stress enhanced the EL, MDA, and H_2_O_2_ concentration, and it enhanced the antioxidant enzymes activities in the rice plants (Figure 4 and Figure 5). The Cr stress may trigger oxidative stress in plants and initiate ROS overproduction in plants [57]. This oxidative stress and overproduction of ROS might disturb the physiological process in plants and ultimately decrease plant growth. The exogenous application of Fe NPs significantly ameliorated the Cr-induced oxidative stress in the rice plants, which was evident by the reduced level of EL, MDA, and H_2_O_2_ and increased antioxidant enzymes activities in plants under Cr stress (Figure 4 and Figure 5). The Fe NPs can decrease ROS production and improve the activities of the enzyme in the rice plants under Cr stress by improving the chlorophyll contents and better transpiration [58]. The inoculation of rice plants with bacteria also significantly decreased the EL, MDA, and H_2_O_2_ contents and increased antioxidants enzymes activities by detoxifying the chromium. The plants treated with Ag-nano-particle modulated ABA, IAA, and GA production and increased the proline production. Additionally, it also reduced the oxidative stress, and augmented bacteria detoxified the toxic effect of Pb, Cd, and Ni on maize plants [59]. Our findings suggest that foliar application of Fe NPs combined with bacterial inoculation can help rice plants cope with Cr stress by regulating the plant’s defense system.

The build-up of toxic metals in the plant root and shoots has a deleterious effect on plant growth. The incorporation of these plants into the food chain may harm human health. The finding of our study suggested that the accumulation of Cr in the rice plant was dose-dependent in the absence of Fe NPs and bacterial inoculation (Figure 6). These findings were comparable to previous studies indicating the increased build-up of metals in the plants with increasing doses of heavy metals [48,60]. The foliar application of Fe NPs resulted in decreased accumulation of Cr in the root, shoot, and grains despite the increased dose of chromium. Iron is an essential nutrient required for the healthy growth, development, and production of the plant. The foliar application of Fe at the nanoscale increased the bioavailability compared to the bulk supply of micronutrients [61]. Thus, the application of Fe NPs might enhance the mineral contents in the plants as compared to the control and reduced the Cr uptake by the plants. Fe NPs as iron oxides have been reported to increase the yield parameters of different crops such as soybean and white and green beans by increasing the nutrients contents in the plants [62,63].

Similarly, the carbon nanoparticles combined with nitrogenous fertilizers improved the yield of the rice crops grown on saline–alkali soil by strengthening the plants to tolerate environmental stress [64]. The inoculation of Cr resistant bacteria and Fe NPs further reduced the Cr accumulation in roots, shoots, and grains. It might be attributed to the ability of bacteria to transform the hexavalent chromium into a less toxic trivalent form. Bacteria also can eliminate the toxic metals through their metabolic process and reduce the bioavailability of toxic metal to plants by adsorption to metals ions [59]. Many researchers reported similar findings where inoculation of plants improved the plants’ growth and reduced the bioavailability of heavy metals and accumulation in the plants [65,66,67]. The increasing dose of Fe NPs increased the accumulation of Fe NPs in the rice grains in the absence of chromium. However, the increasing dose of Cr reduces the Fe NPs accumulation in the rice grains. The nanoparticles can sorb the heavy metals and alleviate the toxicity of the metal as sulfidized nano zero valent iron nZVI (FeSSi) sorb cadmium (Cd) from an aqueous medium and alleviated the Cd toxicity by removing 80% of the cadmium in the first hour [68].

## 5. Conclusions

Chromium stress drastically reduced rice plant growth and photosynthesis and increased plants’ EL, MDA, and H_2_O_2_. Fe NPs improved photosynthesis and carotenoid contents, reduced oxidative stress, and thus improved the plants’ growth and yield by reducing the chromium toxicity in the plants under stress. In addition, the inoculation of plants with *S. aureus* L. further enhanced plant growth by decreasing the Cr toxicity by transforming Cr^6+^ to Cr^3+^ and reducing the bioavailability of Cr to rice plants. Finally, the integrated application of Fe NPs and *S. aureus* L. has proven a promising approach for alleviating Cr toxicity from contaminated soil and enhancing the plants’ ability to endure metals-induced toxicity. Further work should focus on the standardization of the application of NPs in the crops to eliminate the contamination effect of nanoparticles. There is a need to identify and isolate the metal-resistant bacteria with unique abilities to perform efficiently with metal NPs to alleviate the metals induced toxicity in plants.

## Figures and Tables

**Table 1:** Physico-chemical analysis of soil samples from the field of the University of Agriculture, Faisalabad, Pakistan.

Soil	Units
Textural Class	Sandy Clay Loam
Sand	63.7%
Silt	14.4%
Clay	21.9%
pH	7.71
EC	1.93 dS m^−1^
HCO_3_^−1^	3.1 mmol L^−1^
Total nitrogen	0.06%
Available P	2.7 mg kg^−1^
K^+^	0.08 mmol L^−1^
Cl^−1^	5 mmol L^−1^
Ca^+2^ + Mg^+2^	14.34 mmol L^−1^
Available Cd	0.06 mg kg^−1^

## Data Availability

The data presented in the manuscript is the sole data and no other data is linked with this data.
